# Optimizing INFOGEST Digest Conditioning for Reliable In Vitro Assessment of Nutrient Bioavailability Using Caco-2 Cell Models

**DOI:** 10.3390/nu18020339

**Published:** 2026-01-21

**Authors:** Giulia Camporesi, Carlo Canzian, Alessandra Bordoni

**Affiliations:** 1 Interdepartmental Centre for Industrial Agri-Food Research (CIRI), University of Bologna, Piazza Goidanich 60, 47521 Cesena, Italy; giulia.camporesi9@unibo.it; 2 Department of Agricultural and Food Sciences (DISTAL), University of Bologna, Piazza Goidanich 60, 47521 Cesena, Italy; carlo.canzian2@unibo.it

**Keywords:** Caco-2 cells, cell viability, digest conditioning, digestion, epithelial barrier integrity, INFOGEST, in vitro bioavailability

## Abstract

**Background/Objectives**: Assessing the bioavailability of nutrients and bioactive compounds in vitro commonly relies on coupling standardized gastrointestinal digestion models with intestinal epithelial cell systems. However, digests produced using static digestion protocols such as INFOGEST often impair epithelial barrier integrity, limiting their direct application to intestinal models and reducing reproducibility across studies. **Methods**: This work systematically compared five commonly used digest conditioning strategies, including acidification, centrifugation, rapid freezing, and ultrafiltration using 10 kDa and 3 kDa molecular weight cut-off membranes, to identify the approach that best preserves intestinal epithelial viability and barrier function while enabling exposure at physiologically relevant concentrations. INFOGEST digests of yogurt were initially evaluated, followed by validation using biscuit and canned mackerel digests. Cell viability and monolayer integrity were assessed in differentiated Caco-2 cells using MTT assay and transepithelial electrical resistance (TEER) measurements. **Results**: Among the tested approaches, ultrafiltration using 3 kDa membranes consistently preserved epithelial viability and barrier integrity at a 1:10 dilution across all food matrices, whereas other conditioning methods failed to maintain TEER despite acceptable cell viability. At lower dilutions, food-dependent effects emerged, highlighting the importance of matrix-specific evaluation. **Conclusions**: These findings identify 3 kDa ultrafiltration as an effective and minimally invasive strategy to improve the compatibility of INFOGEST digests with intestinal cell models. By enabling reproducible exposure conditions that preserve epithelial integrity, this approach supports more reliable in vitro assessment of nutrient bioavailability and contributes to methodological standardization in nutrition research.

## 1. Introduction

In food and nutrition sciences, two concepts are gaining increasing importance: bioaccessibility and bioavailability. Bioaccessibility is the fraction of an ingested compound that is biochemically and/or physically released from the food matrix during digestion and becomes solubilized in gastrointestinal fluids in an absorbable form, thus becomes potentially available for absorption; bioavailability is the fraction of the ingested compound that is actually absorbed, reaches the systemic circulation (relative bioavailability), and then the target cells, tissues, or organs in the body [1].

Understanding the bioavailability of nutrients and bioactive compounds is essential for accurately linking food composition to physiological effects. While the chemical composition of foods provides valuable information, only the fraction of a compound that becomes bioaccessible during digestion and is subsequently absorbed through the intestinal epithelium can exert biological activity. Consequently, the assessment of bioaccessibility and bioavailability has become a central aspect of contemporary nutrition research.

Although the mechanisms that make a food component bioaccessible and bioavailable vary depending on the nature of the component itself (macronutrients, micronutrients and bioactives), they nevertheless represent a critical bottleneck for biological activity. It is therefore evident that it is necessary to verify the bioaccessibility and bioavailability of nutrients and bioactives before evaluating the effects of a food.

In vitro approaches are increasingly employed to investigate these processes, and several models have been developed to measure bioaccessibility in vitro. Among them, INFOGEST [2] is a widely recognized standardized static model [3] that shows good correlation with in vivo animal feeding studies [4] and is extensively used to estimate nutrient bioaccessibility across different food matrices.

In vitro assessment of relative bioavailability is much more difficult, and currently in vivo methods, which rely on measuring a compound of interest in blood, represent the “gold standard.” However, these studies present ethical issues and are time- and resource-intensive [4]. To assess the absorption of bioaccessible compounds, cellular models are frequently used, including both cultured intestinal cells (in vitro models) and intestinal fragments (ex vivo models). Caco-2 cells, human cells that derive from a colorectal adenocarcinoma and spontaneously differentiate into enterocyte-like cells at confluence [5] are among the most used models, sometimes in combination with mucus-secreting HT29-MTX cells. To evaluate absorption and metabolism through intestinal cells, Caco-2 cells are grown on cell culture inserts that allow for an apical chamber (intestinal lumen) and a basolateral chamber representing the blood compartment.

To realistically simulate the in vivo situation, the cell models are combined with in vitro digestion models. This combination of the two in vitro methodologies, however, poses a major problem because the digest obtained through in vitro static digestion protocols, such as INFOGEST, contains digestive enzymes and bile salts at concentrations that can impair viability and barrier integrity in epithelial cell models such as Caco-2 [6]. Osmolality can also be a problem, since an increase in osmolality caused by food components in the digesta can enhance paracellular permeability of the Caco-2 monolayer [7].

Various approaches have been developed to make the INFOGEST digest compatible with cell cultures, as reviewed by Kondrashina et al. [6]. These include dilution, heat inactivation, enzyme inhibition, filtration, centrifugation, pH, and dialysis, each with distinct advantages and disadvantages. Currently, no consensus protocol has been established. This lack of standardization hampers reproducibility and complicates comparisons between studies.

The present study addresses this methodological gap by systematically comparing five commonly used digest conditioning strategies with the aim of identifying an approach that preserves intestinal epithelial integrity while enabling exposure at physiologically relevant concentrations. To this end, by examining the most frequently used conditioning approaches, we selected the most physiological and least time-consuming ones [6]. All experiments were performed in parallel with a blank digest (digestion without any food) conditioned with the same procedure, to allow distinguishing between potential toxic effects of the intrinsic digestive juices and the food component. We hypothesized that digest conditioning would differentially improve the biocompatibility of INFOGEST digests, enabling identification of a procedure that maintains Caco-2 viability and barrier function and supports discrimination between effects of digestive fluids and food-derived components.

By focusing on multiple food matrices, this work seeks to provide practical guidance for improving the reliability and comparability of in vitro nutrient bioavailability studies.

## 2. Materials and Methods

### 2.1. Chemicals and Materials

Dulbecco’s modified Eagle’s medium (DMEM) high glucose, DMEM without phenol red, penicillin, streptomycin, non-essential amino acids, and Fetal Bovine Serum (FBS) were obtained from Thermo Fisher Scientific (Waltham, MA, USA). Dulbecco’s phosphate-buffered saline (DPBS) was bought from Euroclone (Milan, Italy). Trypan blue was provided by Bio-Rad Laboratories (Hercules, CA, USA). Cells were seeded on Transwell 12-well plates (Corning Incorporated, New York, NY, USA). All other chemicals and solvents were of the highest analytical grade from Sigma-Aldrich Co. (St. Louis, MO, USA).

### 2.2. Food Samples

Three different commercial foods were considered: whole yogurt, biscuits, and canned mackerel. All food samples were purchased at a local market. The chemical composition of the foods under examination, as reported in the nutritional label, is shown in Supplementary Table S1.

### 2.3. In Vitro Digestion

Five grams of each food were in vitro digested according to the INFOGEST protocol [2]. Each time, a blank digestion was carried forward using the same procedure, replacing the food sample with distilled water (5 mL). The digestion was carried out at 37 °C and included a 2 min oral phase at pH 7, a 120 min gastric phase at pH 3, and a 120 min intestinal phase at pH 7. During the different phases, simulated salivary fluid (containing 75 U/mL of amylase), simulated gastric fluid (containing 2000 U/mL of pepsin), and simulated pancreatic fluid (containing 160 mM of bile and an amount of pancreatin such that the trypsin activity in the final mixture was 100 U/mL) were added, respectively. At the end of the intestinal digestion, samples were centrifuged at 4500× *g* for 10 min at 4 °C, and the supernatants were aliquoted into different tubes to be subsequently treated with the different conditioning protocols.

### 2.4. Digest Conditioning

To mitigate the reported toxic effects [6], yogurt digests were conditioned with different protocols: (i) acidification to pH 2.0 using 1 M HCl (adapted from Cilla et al., [8]); (ii) centrifugation at 14,000× *g* for 60 min at 4 °C [9]; (iii) ultrafiltration at 4000× *g* for 60 min at 4° C using 10 kDa and (iv) 3 kDa cut-off membranes (Vivaspin 20) [10,11]; (v) rapid freezing in liquid nitrogen for 35 s [12]. After treatment, digests were sterile-filtered (0.22 µm) and stored at −80 °C until use. Biscuits and canned mackerel digests were conditioned with ultrafiltration on 10 kDa and 3 kDa membranes, sterile-filtered (0.22 µm), and stored at −80 °C until use.

### 2.5. Caco-2 Cell Culture and Supplementation

Experiments were carried out using Caco-2 cells European Collection of Authenticated Cell Cultures (ECACC) cultured in Dulbecco’s modified Eagle’s medium (DMEM) with 4.5 g/L glucose, supplemented with 10% heat-inactivated fetal bovine serum (FBS), 1% L-glutamine, 1% non-essential amino acids (NEAAs), and 1% penicillin/streptomycin, at 37 °C, 5% CO_2_, and 95% humidity. The medium was refreshed every 2 days, and cells were passaged when reaching 80% confluence. Caco-2 cells were used between passages 24 and 38 for all experiments. For experiments, cells were seeded on Transwell 12-well plates (Corning Incorporated, New York, NY, USA) at 1 × 10^5^ cells/mL and differentiated for 21 days. Differentiation was confirmed by transepithelial electrical resistance (TEER) measurement using a Millicell ERS apparatus (Millipore, Burlington, MA, USA). TEER values between 1000 and 2000 Ω·cm^2^ were considered acceptable for the assay. Indeed, in Caco-2 intestinal monolayers, TEER values have been observed from ~600 up to ~2800 Ω·cm^2^ under various conditions, with values above ~1000 Ω·cm^2^ consistent with established barrier formation and used as an integrity benchmark in the literature. Additionally, modern TEER measurement systems are validated across ranges up to ~2000 Ω·cm^2^, further supporting the use of this range as acceptable for assessing intact epithelial resistance [13,14].

After complete differentiation, apical and basal compartments were washed twice with 1 mL phosphate-buffered solution (PBS) and supplemented with the conditioned digests at decreasing dilutions (from 1:40 to 1:5) in serum-free, phenol red-free DMEM for 2–4 h. Controls received only DMEM. All results are the mean ± SD of at least two independent experiments.

### 2.6. Cytotoxicity Evaluation

Cytotoxicity was assessed by evaluating cell viability and monolayer integrity. Cell viability was measured with the methylthiazolyldi-phenyl-tetrazolium bromide (MTT) colorimetric assay [15] using a Tecan Infinite F200 microplate reader (Tecan, Männedorf, Switzerland), and it was expressed as a percentage of control cells (assigned as 100%). Monolayer integrity was evaluated by transepithelial electrical resistance (TEER) measurement using a Millicell ERS apparatus (Millipore, Burlington, MA, USA). TEER was measured immediately before and immediately after the end of the treatment, and percent TEER was calculated as

$$\mathrm{TEER variation}\;(\%)=\frac{\mathrm{TEER before treatment}}{\mathrm{TEER after treatment}}\times 100$$

TEER variation in the treated sample was then normalized to control TEER variation.

$$\mathrm{Normalized TEER}=\frac{\mathrm{TEER\% (treatment)}}{\mathrm{TEER\% (control)}}\times 100$$

### 2.7. Statistical Analysis

Statistical analysis was performed on at least 2 replicates from independent experiments using one-way ANOVA followed by Dunnett’s post hoc test, considering *p* < 0.05 as significant.

## 3. Results

In this study, each experiment was planned based on the results of the previous experiment. Starting with the 1:40 dilution, cells were supplemented with yogurt and corresponding blank digests treated with the different conditioning methods. Supplementation was considered cytotoxic if cell viability and/or normalized TEER were less than 80% of control cells, a conservative threshold commonly applied in in vitro studies to exclude cytotoxic effects and avoid confounding functional readouts [10,15,16,17]. In this case, the corresponding supplemented digest was excluded, and subsequent experiments were repeated under the same experimental conditions using the remaining conditioning treatments at lower dilutions and/or in combination.

**Figure 1:**
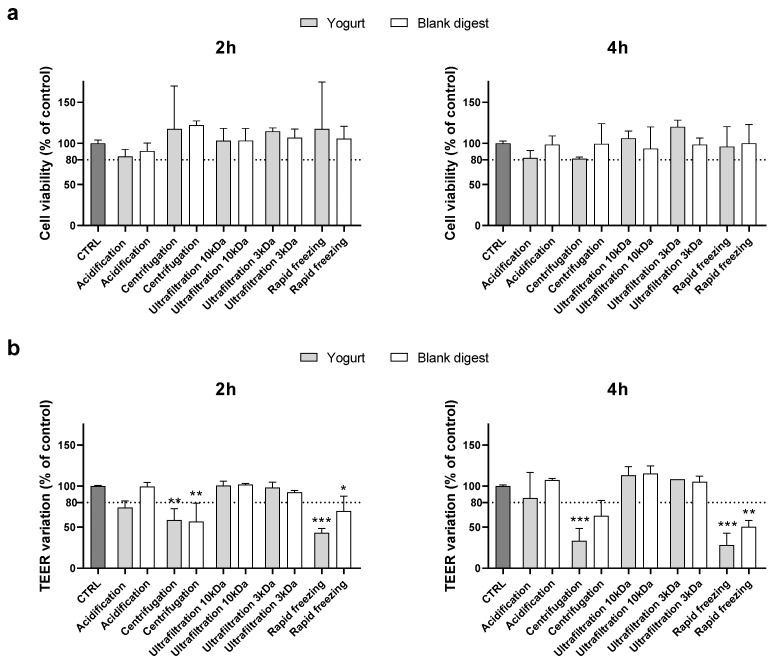
Cytotoxicity after supplementation with 1:30 digests. Cell viability (panel (**a**)) and TEER (panel (**b**)) were evaluated after 2 and 4 h supplementation as reported in Methods section. Data are expressed as percent of control value (assigned as 100%) and are means ± SD of 2 independent experiments. Statistical analysis was performed using one-way ANOVA followed by Dunnett’s post hoc test (*** *p* < 0.001; ** *p* < 0.01; * *p* < 0.05 vs. control cells). CTRL = control.

Supplementing cells with the 1:40 dilution of the conditioned digests, no cytotoxic effects were evidenced, and experiments were repeated using a lower dilution (1:30). As shown in Figure 1, the MTT assay did not evidence any effect on cell viability, but centrifugation and rapid freezing in liquid nitrogen did not exceed the TEER cut-off, either for the food or the blank digests after 2 and 4 h supplementation.

Therefore, in the following experiment, centrifugation and rapid freezing in liquid nitrogen were excluded, and yogurt and blank digests detoxified with the other treatments were supplemented at 1:20 dilution. In this experiment, since acidification showed borderline results when supplemented at 1:30 dilution, it was also combined with ultrafiltration.

**Figure 2:**
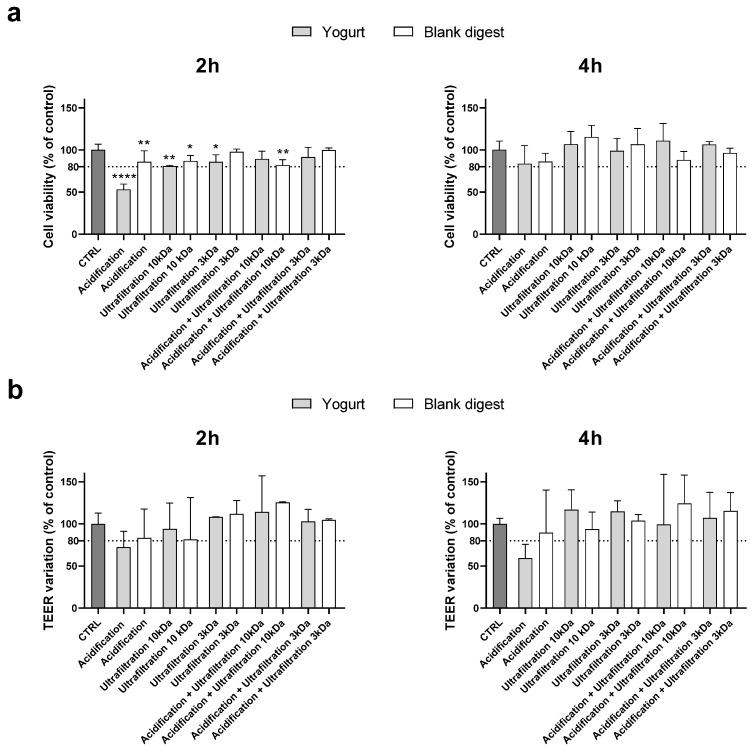
Cytotoxicity after supplementation with 1:20 digests. Cell viability (panel (**a**)) and TEER (panel (**b**)) were evaluated after 2 and 4 h supplementation as reported in the Method section. Data are expressed as percent of control value (assigned as 100%) and are means ± SD of 2 independent experiments. Statistical analysis was performed using one-way ANOVA followed by Dunnett’s post hoc test (**** *p* < 0.0001; ** *p* < 0.01; * *p* < 0.05 vs. control cells). CTRL = control.

As shown in Figure 2, cell viability was less than 80% of the control value after 2 h of supplementation with the acidified digest, which also caused a TEER variation that did not exceed the cut-off. Although TEER measurements revealed high variability within the same treatment, acidification was clearly unable to detoxify the digests. The combination of acidification and ultrafiltration did not prove to be a successful strategy as it did not provide synergistic protection compared to ultrafiltration alone. Therefore, considering that acidification can substantially alter the chemical composition of the food digest, further experiments were conducted using only ultrafiltered digests.

**Figure 3:**
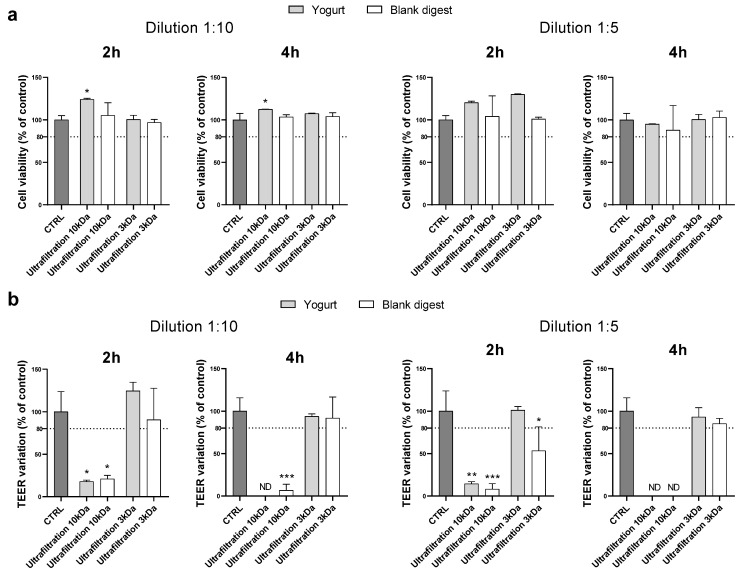
Cytotoxicity after supplementation with 1:10 and 1:5 yogurt and blank digests. Cell viability (panel (**a**)) and TEER (panel (**b**)) were evaluated after 2 and 4 h supplementation of 1:10 and 1:5 digests as reported in the Method section. Data are expressed as percent of control value (assigned as 100%) and are means ± SD of 2 independent experiments. Statistical analysis was performed using one-way ANOVA followed by Dunnett’s post hoc test. (*** *p* < 0.001; ** *p* < 0.01; * *p* < 0.05 vs. control cells). CTRL = control, ND = not detectable.

By supplementing cells with digests at a 1:10 dilution, ultrafiltration through 10 kDa membranes proved to be an effective method to make the digest compatible with Caco-2 when cytotoxicity was assessed by MTT, but the TEER decreased strongly after either 2 or 4 h of supplementation. Ultrafiltration on 3 kDa membranes effectively protected cells from toxicity. Indeed, both MTT and TEER results exceed the chosen cut-off (Figure 3).

Experiments were repeated using the 1:5 dilution, and we observed similar results (Figure 3). Therefore, ultrafiltration with 3 kDa membranes was found to be the most suitable method to condition the yogurt digest, which did not appear cytotoxic even at very low dilution (1:5), at least in the experimental conditions reported.

To evaluate whether the results obtained were related to yogurt digest only or could be generalized to other types of food, digests of biscuits and canned mackerel were supplemented to Caco-2 cells after ultrafiltration through 10 and 3 kDa membranes.

**Figure 4:**
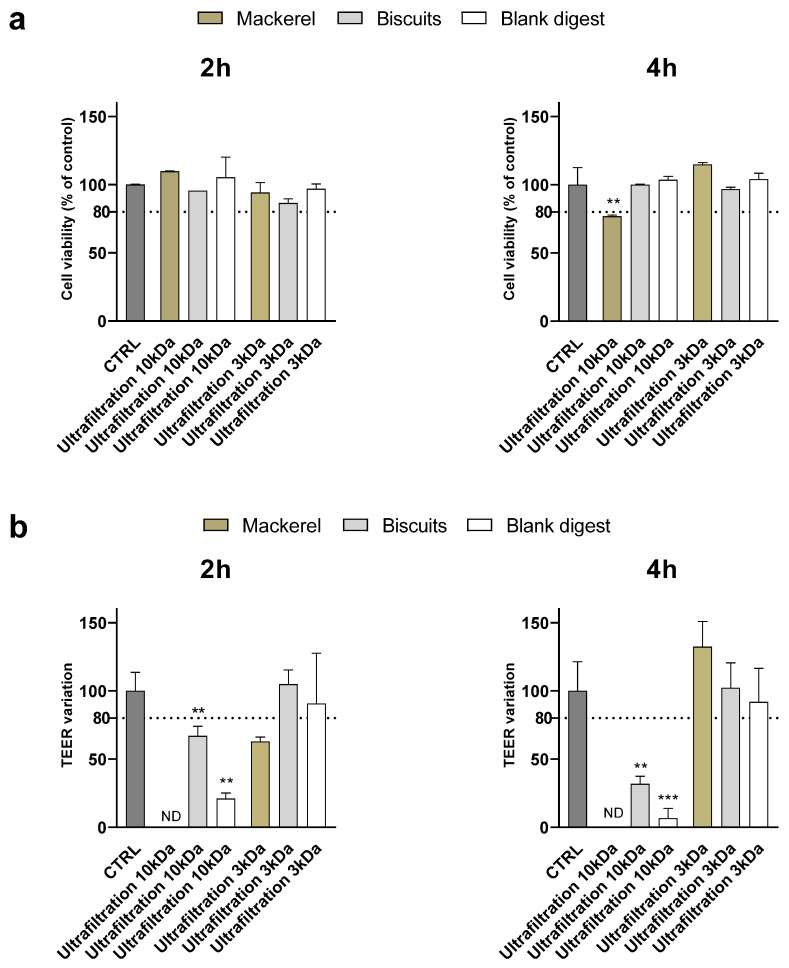
Cytotoxicity after supplementation with 1:10 canned mackerel and biscuit digests. Cell viability (panel (**a**)) and TEER (panel (**b**)) were evaluated after 2 and 4 h supplementation of 1:10 digests as reported in the Method section. Data are expressed as percent of control value (assigned as 100%) and are means ± SD of 2 replicates in each condition. Statistical analysis was performed using one-way ANOVA followed by Dunnett’s post hoc test comparing (i) mackerel and corresponding blank vs. control cells; (ii) biscuits and corresponding blank vs. control cells (*** *p* < 0.001; ** *p* < 0.01). CTRL = control; ND = not detectable.

Similarly to what was observed for the yogurt digest, at 1:10 dilution, ultrafiltration through 10 kDa membranes did not preserve the integrity of the monolayer—assessed by TEER measurement—in any of the digests, although no cytotoxicity was detected by the MTT assay, except in mackerel, after 4 h of supplementation (Figure 4). Ultrafiltration through 3 kDa membranes efficiently counteracted the toxicity of the biscuit digest, while monolayer integrity was disrupted after 2 h, but not 4 h, supplementation with mackerel digest.

**Figure 5:**
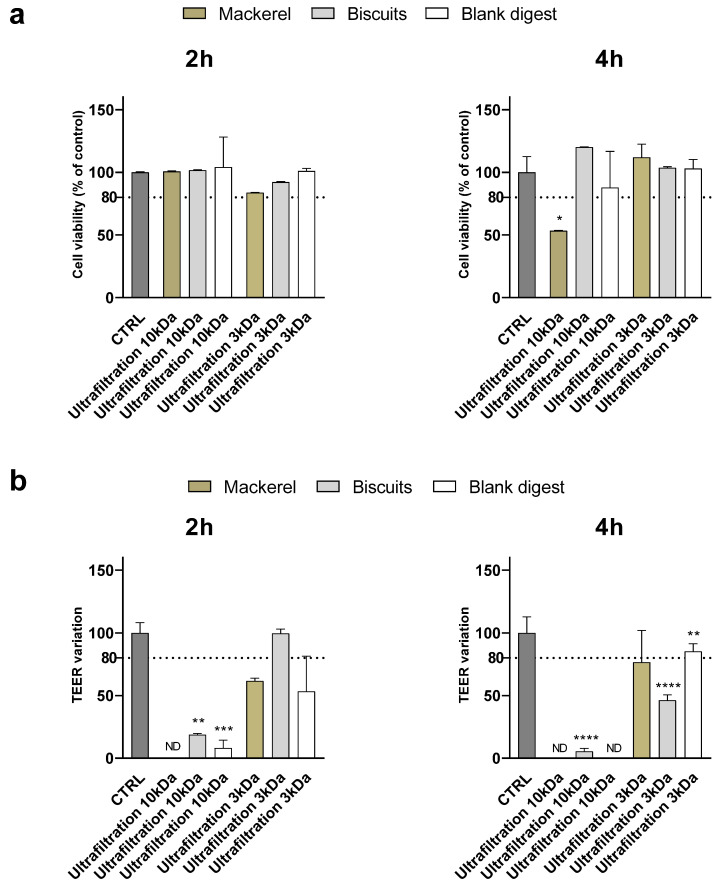
Cytotoxicity after supplementation with 1:5 biscuits and canned mackerel digests. Cell viability (panel (**a**)) and TEER (panel (**b**)) were evaluated after 2 and 4 h supplementation of 1:5 digests as reported in the Method section. Data are expressed as percent of control value (assigned as 100%) and are means ± SD of 2 replicates in each condition. Statistical analysis was performed using one-way ANOVA followed by Dunnett’s post hoc test comparing (i) mackerel and corresponding blank vs. control cells; (ii) biscuits and corresponding blank vs. control cells (**** *p* < 0.0001; *** *p* < 0.001; ** *p* < 0.01; * *p* < 0.05). CTRL = control; ND = not detectable.

At the 1:5 dilution, although cell viability was significantly affected only by the ultrafiltered mackerel digest (<10 kDa) after 4 h of supplementation, the TEER variation showed an alteration of the monolayer integrity after supplementation of all digests except the ultrafiltered biscuit < 3 kDa after 2 h (Figure 5). Supplementary Tables S2 and S3 summarize and compare the effectiveness of digest ultrafiltration across the various food matrices studied.

Lower digest dilutions (<1:5) were not considered for further experiments, since the most effective treatment (ultrafiltration < 3 kDa) did not meet the TEER acceptance criterion for all samples, suggesting a food-specific effect.

**Figure 6:**
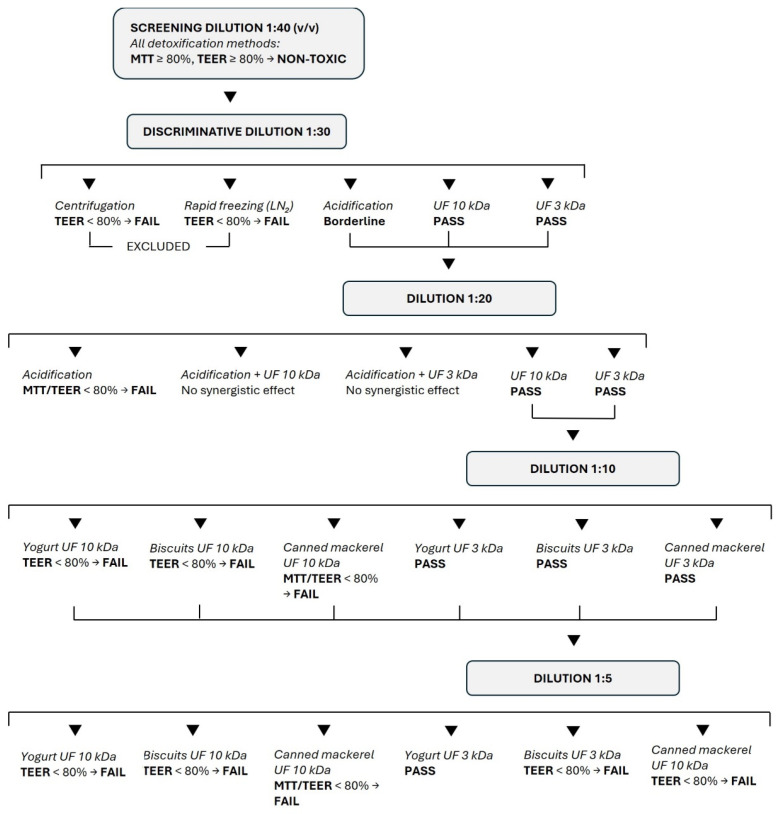
Flow chart of the experimental design and the obtained results.

The results of the study are summarized in Figure 6.

## 4. Discussion

Growing awareness of the importance of assessing food bioavailability increases the need for standardized protocols that enable meaningful comparison between in vitro studies. Currently, several inactivation/detoxification methods have been tested, as reviewed by Kondrashina et al. [6], and since each involves modifications to the digest, defining the best method is difficult without a direct comparison. In this study, we detoxified the same yogurt digest obtained with the INFOGEST protocol using five different methods, then supplemented the resulting detoxified samples to Caco-2 cells at decreasing dilutions, with the aim of identifying the method that allowed supplementation at the highest concentration. Procedures such as enzyme inhibition, temperature, and dialysis, considered non-physiological or time-consuming [6], were not tested in the study. A condition involving dilution without digest conditioning was not included, as unconditioned INFOGEST digests are known to impair epithelial viability and barrier integrity even at high dilutions, and therefore do not represent a suitable or interpretable control for intestinal bioavailability studies [6,18,19].

Regardless of the conditioning method, no signs of toxicity were observed when supplementing digests diluted 1:40. The 1:30 dilution, however, discriminated between the different methodologies; in fact, neither centrifugation nor rapid freezing in liquid nitrogen met the viability criteria. At a 1:20 dilution, we also tested the potential synergistic effect of treatments by combining acidification and ultrafiltration on 10 or 3 kDa membranes. Combining the two methodologies did not provide additional benefits, and acidification (pH 2.0) caused extensive protein precipitation, significantly altering the chemical properties of the digest. Therefore, although acidification has been successfully applied in studies targeting specific classes of bioactive compounds, such as polyphenols, allowing supplementation even at lower dilutions (1:13) [8], it was excluded, as it does not preserve the native characteristics of the digest.

Ultrafiltration is a less invasive method than acidification and better preserves the nature and composition of the digests, considering that further studies for which they may be intended usually focus on the presence of small molecules that are not retained by ultrafiltration membranes. We ultrafiltered the digests through 10 and 3 kDa membranes, since the enzymes used in in vitro digestion have molecular weights greater than 10 kDa (porcine pepsin, 41 kDa; pancreatic lipase, 51 kDa; porcine trypsin, 24 kDa; chymotrypsin, 29 kDa; porcine α-amylase, 57 kDa) [20].

Using a 1:10 dilution, it became evident that ultrafiltration on 10 kDa membranes was not sufficient to preserve the integrity of the monolayer, even though cell viability was comparable to that of control cells. This observation highlights the need to go beyond the simple measurement of cell viability before defining the concentration of digest to be supplemented to intestinal cells. Indeed, although cell viability was assessed using the MTT assay, which has been reported to be one of the most sensitive tests for detecting cytotoxicity [15], the results obtained by considering this assessment alone would have been misleading. The integrity of the Caco-2 monolayer should be assessed by evaluating the TEER, a noninvasive technique that measures the impedance between the lumen and the basolateral tissue. Toxic effects can affect the stability of the tight junctions, altering cellular permeability, and monitoring epithelial barrier function by measuring TEER values before and after the supplementation test is essential to consistently assess the bioavailability of a food component [16].

The toxicity of digests after ultrafiltration through 10 kDa membranes leads us to hypothesize that digestive enzymes, although hydrolyzed during digestion, may form smaller (10 to 3 kDa in size) but still active residues. Furthermore, in the INFOGEST in vitro digestion protocol, bile acids are used in the intestinal phase to emulsify the lipid content of the food. While single bile acids are relatively small, in an aqueous environment they form micelles that can reach dimensions in the range of 3–6 (simple micelles) to 20 nm (mixed micelles including phospholipids, fatty acids, etc.) [21]. Although bile salt micelles do not have a “kDa mass” because they are not single molecules, but dynamic aggregates of many amphipathic molecules, based on the average molecular mass of a conjugated bile acid and the estimated number of molecules per micelle, it can be hypothesized that most bile acid micelles do not cross the 3 kDa membrane, but that smaller micelles might be present in the <10 kDa ultrafiltered digest, explaining the difference in cytotoxicity parameters between the two.

Ultrafiltration < 3 kDa allowed supplementation without detectable impairment of cell viability or barrier integrity for up to 4 h at a 1:10 dilution, regardless of the type of food digested. The greater effects on TEER after 2 h of supplementation observed in mackerel may be due to mechanical stress from washing or medium changes that supplementation entails. Importantly, a further reduction in dilution (1:5) showed significant differences between the tested foods. Indeed, at this dilution, the ultrafiltered (<3 kDa) blank and yogurt digests were not toxic, suggesting that ultrafiltration eliminated toxic components bound to digestive fluids required for in vitro digestion, but that small, potentially cytotoxic molecules may be released during the digestion of some foods. We acknowledge that ultrafiltration at 3 kDa may reduce the recovery of larger digestion-derived bioactive peptides; however, gastrointestinal digestion generally produces peptide mixtures enriched in low-molecular-weight sequences, many of which fall below this cut-off and are most relevant for epithelial exposure and absorption [22]. Larger peptides (>3 kDa), although potentially bioactive, are less likely to be absorbed intact and typically undergo further hydrolysis at the brush border prior to uptake. Thus, while some larger peptides may be excluded, 3 kDa ultrafiltration is expected to preferentially retain peptides with higher physiological relevance for in vitro bioavailability studies. As an example, simulated gastrointestinal digestion of yogurt proteins, particularly caseins and whey proteins, generates a complex peptide mixture that is strongly enriched in low-molecular-weight peptides, with many well-characterized yogurt-derived bioactive sequences—such as angiotensin-converting enzyme (ACE)-inhibitory, antioxidant, and immunomodulatory peptides— consistent with molecular weights below 3 kDa and often corresponding to oligopeptides of fewer than 20 amino acids [23,24].

Some limitations of the present study should be acknowledged. pH, osmolality, and residual bile salt concentrations were not directly measured in the conditioned digests, although all these factors are known to influence epithelial barrier integrity in Caco-2 models [7,25,26]. However, the study was designed as a functional, side-by-side comparison of conditioning strategies using complementary endpoints—cell viability and TEER—that are highly sensitive to these stressors and directly relevant for subsequent bioavailability assessments. In addition, a limited number of biological replicates was used for selected experimental conditions, reflecting the complexity, duration, and high resource demands of the coupled in vitro digestion–epithelial barrier assays. Nevertheless, all key trends were reproducible across independent experiments and consistent across food matrices. Statistical analyses were therefore adapted to the experimental design, and conclusions are primarily based on consistent comparative trends observed across conditioning methods rather than on absolute effect sizes.

This study advances existing work on the biocompatibility of in vitro digests with intestinal epithelial models by providing a systematic, side-by-side comparison of commonly used INFOGEST digest-conditioning strategies applied to identical digestion outputs. Unlike many previous studies in which conditioning approaches are evaluated individually or selected empirically, the present work directly contrasts multiple methods across decreasing digest dilutions using both cell viability and epithelial barrier integrity as quantitative endpoints. The inclusion of identically conditioned blank digests further allows discrimination between cytotoxic effects arising from digestive fluids and those derived from food components. Importantly, the combined evaluation of MTT and TEER measurements demonstrates that preserved cell viability does not necessarily imply maintained epithelial barrier function, underscoring the need to incorporate barrier integrity as a critical criterion when coupling digestion models with intestinal cell systems.

Although the present study included food matrices differing in composition, extension of this approach to additional food categories, such as highly lipid-rich or polyphenol-rich matrices, will be required to further explore matrix-specific effects and refine general methodological recommendations.

## 5. Conclusions

In vitro approaches are increasingly employed to assess the bioavailability of nutrients and bioactive compounds, offering clear advantages in terms of ethical considerations, experimental control, and mechanistic insight. However, meaningful bioavailability assessment requires intestinal epithelial models that preserve barrier integrity, as impairment of epithelial viability or tight-junction function may lead to overestimation of absorption. When simulated digestion is coupled with epithelial models, components of the digestive fluids—rather than the food itself—can compromise cell viability and barrier function, and this issue is often addressed through extensive dilution, limiting physiological relevance and comparability among studies. The present study demonstrates that ultrafiltration using a 3 kDa molecular weight cut-off represents an effective and minimally invasive strategy to condition INFOGEST digests for application to intestinal epithelial models. This approach enabled exposure at a 1:10 dilution without detectable impairment of cell viability or epithelial barrier integrity across multiple food matrices, while lower dilutions revealed food-dependent effects, underscoring the need for preliminary compatibility testing. Despite variability related to digestion protocols, food matrices, and cell models, the systematic comparison presented here provides practical guidance to improve the reproducibility and physiological relevance of in vitro nutrient bioavailability studies. Some limitations should be acknowledged, including peptide partitioning after ultrafiltration. Future studies should integrate digest conditioning with quantitative physicochemical characterization and peptidomics-based bioactivity analyses of permeate and retentate fractions to clarify the contribution of larger digestion-derived bioactive peptides. Furthermore, translational research should aim to validate these findings using more complex models, such as co-cultures of intestinal epithelial cells and microbiota bacteria (colon models), to understand the impact of conditioning on microbial fermentation and metabolism. Integrating these standardized methodologies with absorption assays in in vivo animal models will be essential to confirm the physiological relevance of these results and further support methodological harmonization in nutrition research.

## Data Availability

The original data presented in the study are openly available in Zenodo at https://doi.org/10.5281/zenodo.17978436.

## Supplementary Materials

The following supporting information can be downloaded at: https://www.mdpi.com/article/10.3390/nu18020339/s1, Table S1. Nutritional composition (g/100 g) of the in vitro digested food items. Table S2. Effectiveness of inactivation methods in different foods on viability (% of control). Table S3. Effectiveness of inactivation methods in different foods on TEER variation (% control).
