# Supplementary material for: Optimizing INFOGEST Digest Conditioning for Reliable In Vitro Assessment of Nutrient Bioavailability Using Caco-2 Cell Models
Source: Nutrients. 2026 Jan 21;18(2):339. doi: 10.3390/nu18020339 (PMC12844764; doi:10.3390/nu18020339)
Supplement: Supplementary file 1 [file nutrients-18-00339-s001.zip › Supplementary Table S1.pdf]

Supplementary Table S1. Nutritional composition (g/100 g) of the in vitro digested food items.

	Kcal	Total Fat	Saturated Fat	Carbohydrates	Sugars	Protein	Fiber	Salt
Yogurt	67	3.5 g	2.4 g	4.8 g	4.8 g	4 g	0 g	0.1 g
Canned Mackerel	141	4.5 g	1.2 g	0 g	0 g	25 g	0 g	0.93 g
Biscuits	483	22 g	10 g	60.4 g	23.4 g	7.6 g	6.3 g	0.43 g
