# Supplementary material for: Optimizing INFOGEST Digest Conditioning for Reliable In Vitro Assessment of Nutrient Bioavailability Using Caco-2 Cell Models
Source: Nutrients. 2026 Jan 21;18(2):339. doi: 10.3390/nu18020339 (PMC12844764; doi:10.3390/nu18020339)
Supplement: Supplementary file 1 [file nutrients-18-00339-s001.zip › Supplementary Table S2.pdf]

Supplementary Table S2. Effectiveness of inactivation methods in different foods on viability (% of control)

		Blank of digestion	Yogurt	Canned Mackerel	Biscuits
			**Dilution 1:10**		
Ultrafiltration 10 kDa	2h	105.33 ± 14.87	124.08 ± 1.34	109,78 ± 0,19	95,57 ± 0,03
	4h	98.78 ± 7.81	112.51 ± 0.05	76,77 ± 0,96	99.99 ± 0.49
Ultrafiltration 3 kDa	2h	96.96 ± 3.48	100.54 ± 5.95	94,20 ± 7,14	86,50 ± 3,03
	4h	98.94 ± 3.46	107.41 ± 0.61	114.81 ± 1.32	96,65 ± 1,52
			**Dilution 1:5**		
Ultrafiltration 10 kDa	2h	104.19 ± 24.03	120.39 ± 1.46	100,66 ± 0,47	101,67 ± 0,35
	4h	84.27 ± 30.51	95.10 ± 0.25	53,35 ± 0,25	120,1 ± 0,20
Ultrafiltration 3 kDa	2h	101.06 ± 2.06	102.19 ± 0.02	83,76 ± 0,27	92,12 ± 0,55
	4h	98.02 ± 8.33	100.47 ± 7.48	111,88 ± 10,56	103,58 ±0,91
