# Supplementary material for: Optimizing INFOGEST Digest Conditioning for Reliable In Vitro Assessment of Nutrient Bioavailability Using Caco-2 Cell Models
Source: Nutrients. 2026 Jan 21;18(2):339. doi: 10.3390/nu18020339 (PMC12844764; doi:10.3390/nu18020339)
Supplement: Supplementary file 1 [file nutrients-18-00339-s001.zip › Supplementary Table S3.pdf]

Supplementary Table S3. Effectiveness of inactivation methods in different foods on TEER variation (% control).

		Blank of digestion	Yogurt	Canned Mackerel	Biscuits
			**Dilution 1:10**		
Ultrafiltration 10 kDa	2h	21.01 ± 4.14	17.85 ± 1.67	N.D.*	67,07 ± 7,01
	4h	6.90 ± 6.95	N.D.*	N.D.*	31,80 ± 5,67
Ultrafiltration 3 kDa	2h	90.63 ± 37.04	124.62 ± 10.11	62,85 ± 3,30	104,91 ± 10,47
	4h	95.20 ± 27.77	93.69 ± 2.98	132,4 ± 18,46	102,19 ± 18,36
			**Dilution 1:5**		
Ultrafiltration 10 kDa	2h	8.20 ± 6.32	14.49 ± 2.41	N.D.*	18,67± 1,05
	4h	N. D.*	N.D.*	N.D.*	5,40 ± 2,43
Ultrafiltration 3 kDa	2h	53.38 ± 28.03	101.06 ± 4.20	61,55 ± 2,30	99,58 ± 3,35
	4h	91.73 ± 8.55	93.09 ± 10.85	76,63 ± 25,46	46,16 ± 6,15

* N.D.: not detectable, as the calculated TEER variation (%) resulted in negative values.
